# Pensions for the Clergy—Why Not for Physicians?

**Published:** 1915-06

**Authors:** 


**A Monthly Review of Medicine and Surgery**

EDITOR AND PUBLISHER, DR. A. L. BENEDICT, 228 Summer St., corner of Elmwood Ave., Buffalo, (Address for all communications. Please make personal and telephone calls before 1 P. M.)

Third, in age, on the western continent. Independent, but supports professional organization. News limited mainly to Western N. Y. and Penn.

Subscription $2.00 a year; $3.00 for two years in advance. Changes and errors in address or failure or duplication of delivery, should be reported immediately.

Yearly Volume 70. JUNE, 1915 No. 11

### Pensions for the Clergy—Why Not for Physicians?

The need of systematic provision for disabled and superannuated clergymen has recently been made the subject of a canvass for funds by certain denominations. We happened to hear an address by a member of a church board, in which the statement was made that a number of professions, including medicine, provided for needy physicians and their families. So far as we are aware, this statement is incorrect as applied to medicine but it ought to be true. A study of statistics shows why the problem before the protestant churches is of great magnitude. The three catholic orders of this country, aside from the Episcopal church, had in 1910, 12,711,673 communicants, 17,404 priests and 13,979 churches. This, it will be observed, is an economic arrangement, providing for congregations of approximately a thousand and for an increase in ministers, rather than the multiplication of churches. All the other religious denominations, mainly protestant Christians, had 22,621,097 communicants, 152,749 ministers and 204,168 churches. When one analyses these statistics, it is obvious that there is loss of efficiency in small congregations, closed churches or churches jointly served by a single minister and the average size of a congregation of only about 140—though the relatively greater number of attendants as compared with communicants, in protestant denominations should be borne in mind.

The average physician has a clientele of about 700, though the affiliation and sense of responsibility is much weaker than in the case of the clergy. The close analogy between the spiritual and physical minister, the identity of their social influence and philanthropic activity is often cited by the laity, though mainly on what may be termed auspicious occasions. A systematic campaign for funds, like that now in progress in churches is out of the question, yet undoubtedly, many laymen would be glad to contribute to so worthy a cause. But we feel that the medical profession should secure its members and their dependents against want in their old age or on account of disability. While the fact that the average physician has five times the clientele of the average protestant clergyman does not count for much directly, it ought to reduce the relative number of the superannuated and of those who, on account of inedaquate support during their active life, have been unable to insure themselves and their families against want.

Just how the philanthropy advocated should be financed, is a matter which cannot be settled off hand. One thing seems to us important: Neither old age nor premature disaster should separate the physician from his professional organization. The expense of such organizations is, under present circumstances, one of the first that the physician is likely to cut off. The very fact that, in a period of adversity, dues are still demanded, may be the main explanation of this fact. The fact that professional organization does nothing for the man unable to practice, is another factor. And the fact that professional affiliation is not absolutely requisite for practice is a third reason. We feel strongly that the first step in providing for the relief of superannuated and disabled physicians should consist in making membership a more permanent and more fraternal factor than the annual exchange of dues for the privileges of scientific and social converse. We estimate the sense of honor of the average physician too highly to anticipate that this step would result in any great financial burden upon more fortunate, active practitioners. If anything, we think the financial burden would be diminished by the more general assumption of membership, if each physician felt that the society would stick to him in time of trouble, at least in the negative sense of carrying his membership during such time. It would be a gracious act and one that would involve no expense whatever if each society would place on a special roster, the name of any active member who discontinued practice for any reason, at any age—unless, of course, to engage in some form of activity at variance with the ethical standards of the profession. If anything, such a courtesy might result in the receipt of favors from retired physicians. At any rate, it has always seemed to us abnormal that the death of a venerable, retired physician, should be officially ignored because, for financial reasons, he has not maintained his membership.

Whether the relief of superannuated and disabled physicians and of their dependents should take the form of charity, according to their needs, or of some form of insurance to be accepted in a purely business spirit, is a phase of the problem that must be carefully studied. It may be asked why not leave insurance to insurance companies? Because, in our opinion, the discredit of having a fellow physician in distress is enough to lead us to insure even against the neglect of insurance and certainly against the failure of insurance to insure—a factor of considerable importance. Moreover, while we do not attempt to decide in advance of critical study, between fraternal assistance and insurance calculated according to experience, the former method allows a degree of discrimination according to actual needs, is more elastic and more fraternal.

It may be pointed out that the burden of assistance may be carried in such a way as not to be overwhelming. For instance, religious organizations as well as educational, are supported to a considerable degree by bequests—a form of benevolence which costs the donor nothing and which, as has been well described, leaves the individual in possession of his capital till it is time to surrender it to God who gave it. Administered as a charity, not as insurance, an appeal may properly be made to the laity. But, even from the strictly business standpoint, insurance may be economically carried by the professional organization as by an extrinsic corporation, more so if the avowed purpose is to give aid only to the extent of relieving suffering. So far as temporary disability benefits are concerned, no class is so favorable as physicians in respect to the period of disability claimed, since it is to the interest of the beneficiary to shorten his period of disability as much as possible. As to relief of the superannuated, it may be stated as approximately correct that 10 per cent. of any fund contributed, could be dispensed as an annuity, with profit.

